# Medicine

**Published:** 1894-09

**Authors:** George Parker


### Progress of the Medical Sciences.

### MEDICINE.

The theory and treatment of uræmia have been undergoing considerable discussion of late. At the New York Neurological Society, C. A. Herter argued that the condition, like dropsy, may be due to a variety of causes.[1] These may be infective processes in some cases; in others an accumulation of extractives in the blood, and in a certain class the symptoms he considered to be produced by simple urea poisoning; *i.e.*, urea acting as irritant poison on the gastro-intestinal membrane. Certainly in these cases, marked by much vomiting and nausea, he found numerous patches of congestion, and was able to reproduce both the anatomical and clinical symptoms by injecting urea into the tissues of animals. Urea in excessive amounts can be detected in the blood, and exists in quantities in the fæces. There is an absence of fever and of any increase of toxicity in the urine, all of which help to mark off this type of uræmia from others.

Dr. Dickinson's paper on "Albuminuric Ulceration of the Bowels"[2] may be perhaps regarded as a corroboration of this view. Though a comparatively rare complication of albuminuria, these intestinal hemorrhages and other lesions have been many times noticed. Twenty-five cases were reported by the writer and many others by those who took part in the discussion; so that there was no question of the existence of the affection, but only whether the ulceration was due to inflammation or hemorrhage. In the light of Herter's experiments, the presumption is that this form of ulcerative colitis is due to the erosive action of urea excreted through the intestinal walls. However, the cases of uræmia where gastro-intestinal symptoms are prominent are not a large proportion of the whole, and it must be confessed that there are great difficulties in the way of the usual explanations of other types. It is noticeable that

[1] *Hospital*, vol. xvi., 1894, p. 299. [2] *Med. Rec.*, vol. xlv., 1894, p. 222.

almost the chief effect of renal extract in albuminuria has been said to be the increased toxicity of the urine eliminated.[1] One curious complication has been adduced in favour of that theory of uræmia which relies on brain œdema as an explanation; and that is uræmic hemiplegia. A number of cases are on record quite distinct from those caused by hemorrhages occurring in renal diseases.[2] The sudden hemiplegia following minor uræmic symptoms terminates after a time in complete recovery, or changes to the other side of the body. If death occurs, no lesions can be detected in the brain.

The use of milk diet in kidney disease has been of late years carried to an injurious extent, and a number of observers have recently insisted on its inadequacy, and even on the danger there is in producing uræmia. Its advantages seem to depend on the easily metabolised form of albumen it contains, the diuretic action of the lactose, and the practical disinfection of the intestine which such a diet produces in a few days. There is not, however, sufficient nourishment for adults to be obtained from reasonable quantities of milk if it forms the sole food. Observations show that chronic cases do much better on a light mixed food, of which carbohydrates form a large part. A whole series of papers and discussions have emphasised this view. In acute nephritis, however, the objections have less force, as an increase of fluids, solids, and of urea, with a decrease of albumen, follow upon adherence to a pure milk diet. Accordingly Dr. Ralfe[3] would restrict its use to these acute cases and to those chronic ones where he specially wishes to reduce pulse tension. Dr. Hale White's conclusions were given on pp. 30-1 of the March number of this *Journal*. Dr. Saundby insists that even a reduction of albumen should not be our chief guide in treatment, but rather the patient's general health, since the albumen is no measure of renal disease, nor evidence of a present inflammatory process, nor usually an important drain on the system. Leaving milk to acute attacks, and even then adding farinaceous food, he advises in other forms a sparing amount of butcher's meat, and the avoidance of malt liquors, spirits, and all but weak wines. No beef tea and only moderate quantities of food are permitted, and meat is stopped if there is any threatening of uræmic nature.[4] Dr. Dickinson with similar diets gives in every case quantities of drinks containing much water, even weak broths;[5] in short, the consensus of modern opinion seems to be that in acute forms milk may be made the chief article of food; but in all others a light and varied diet, with care that the skin and bowels are acting properly, gives the best results.

As to other means for the treatment of uræmia, the use of copious saline enemata, or saline hypodermic injections, vene-

[1] *Lancet.*, 1894, vol. i., p. 1405.

[2] *Am. J. M. Sc.*, vol. cvii., 1894, p. 493. [3] *Lancet*, 1894, vol. i., p. 742.

[4] *Med. Ann.*, 1894, p. 133. [5] *Lancet*, 1894, vol. i., p. 317.

section in strong and cupping in weakly patients, and pilocarpine administered with care, are advocated by some American authorities.[1] Urethane is praised for its efficacy in controlling convulsions, and for its safety in preference to chloroform, chloral, and morphia; while the early symptoms are said to be relieved by large doses of carbonate of iron. It should be noted that pilocarpine, besides occasionally causing danger to the lungs, may, if there is much œdema present, actually bring on acute uræmia. In reference to Dr. R. C. M. Page's[2] warm recommendation of veratrum viride and morphia combined for convulsions when there is high tension, we cannot but be struck with the tendency that lingers in some quarters to meet the dangers of high tension by reducing the cardiac force in place of attacking the constriction of the small vessels. The heart is labouring against a dam which prevents arterial blood passing into the capacious venous system, and surely the reasonable plan is to remove the dam by dilating the arterioles by the nitrites, or to employ purgation or even venesection, rather than to aim at weakening the heart, which becomes exhausted of itself only too soon. In a large class of uræmias we find no high tension at any period, but low muttering delirium, and a specially weak heart. We are reminded in fact of the two forms of angina—the one with, and the other without, high tension. The low tension needs cardiac stimulants; but each form equally requires purification of the blood by purgation, saline injections, copious watery drinks, and hot baths. "We cannot wait until we know the exact poisons which are present"; it is necessary to replace the saturated fluids by fresh ones. The question how far we are justified in giving alkalies as diuretics in uric-acid coma is important in view of the risk of convulsions if accumulations of the uric acid are washed out and circulate in the blood. Nor must we forget Roberts's warning against giving sodium salts in gouty kidneys; but the salicylates which eliminate the urates in a harmless form have little value in combating acute uræmia, as N. S. Davis[3] points out. Haig believes that the blood can be cleared at once of an excess of uric acid by giving the iodide of mercury or iron, which is possibly the explanation of the value of carbonate of iron in uræmia. He afterwards administers the salicylates for a considerable period, and checks the consumption of animal food, especially of lamb, veal, liver or kidney, beef tea, strong coffee or tea, and those vegetables which contain an excess of the xanthin group.[4]

Much has been written on the value of piperazine in the excretion of uric acid, and contradictory results obtained. D. D. Stewart, by cautiously giving as much as seventy grains daily, increased the excretion of water considerably in gout and, on combining it with citrate of potash, rapidly got rid of the pains and swellings. Little increase of the uric acid excreted

[1] *Med. Rec.*, vol. xlv., 1894, p. 321. [2] *N. York M. J.*, vol. lix., 1894, p. 586.
[3] *Internat. Clin.*, vol. iii., 1893. [4] *Hospital*, vol. xvi., 1894, p. 53.

was found; but he suggests that it may have passed out a allantoin, or as urea, and the amount of the latter would not be perceptibly increased.[1]

Considerable efforts have been made to unravel the pathogeny of diabetes. Chauveau and Kaufmann, quoted by Williamson in the *Medical Chronicle* for June, hold that the sugar-forming function of the liver is under the influence of an inhibitory centre in the medulla and of an excitor centre in the cord. The pancreas by its internal secretion acts on these centres through the blood as an additional inhibitory force, hence the glycosuria which follows its removal.[2] However, Dr. de Domenicis, of Naples, has tried to prove that it is the intestinal secretion which is the active part of the pancreas,[3] and argues that the blocking of the duct of Wirsung will cause glycosuria; but Hausemann also, speaking at Rome, referred this result to the consequent atrophy of the pancreatic cells; and, indeed, this seems to agree better with the facts detailed by Minkowski. Domenicis's results would, if accepted, show the uselessness of transplantation of slips of pancreas into the peritoneal cavity. Among other remedies, guaiacol and piperazine have each been credited with some influence on the excretion of sugar, while severe glycosuria has certainly followed the administration of thyroid extract.[4] After it was given up, the glycosuria disappeared as quickly as it came. By far the most important contribution to the pathology of diabetes has been made by the publication of Dr. Pavy's lectures. He demands no less than the reversal of current teaching on the physiology of the liver, and in support of his views brings forward the experiments and facts which he has accumulated in thirty years. Carbohydrates, he insists, do not enter the system as such, but are broken down and built up again into proteids and fats. Energy is developed by the oxidation of the carbon contained in proteid molecules, for proteids are true glucoside bodies, and split up readily into sugar, peptone, and other bodies. Uncombined sugar is prevented from entering the body first by the intestinal cells, while any which penetrates is filtered off by the liver, changed to glycogen, and probably into fat. None is normally manufactured by or allowed to pass the liver, and diabetes appears when this occurs. The arterial blood contains no more sugar than the venous, as it should do if sugar is passing from it to the muscles. In short, a proteid acts as the universal carrier of every variety of food to the tissues, and the difficulty seems to be, as a critic in the *British Medical Journal*[5] points out, how enough proteid matter can be found in ordinary diet to unite with carbohydrate

[1] *Therap. Gaz.*, vol. xviii., 1894, p. 86.

[2] *Compt. rend. Soc. de biol.*, Feb. and Mar., 1893.

[3] *Med. Rec.*, vol. xlv., 1894, p. 503. [4] *Brit. J. Dermat.*, vol. vi., 1894, p. 177.

[5] 1894, vol. ii., p. 86.

elements. However, the chief discussion will centre upon the correctness of the statements which he has alleged with such clearness and originality.

The nervous origin of pyrexia has been discussed both at Rome and Bristol; but it can hardly be said that many new facts have been brought forward. Still, it is important to have emphasis laid on the primary fact that pyrexia may be produced by nerve lesions and mental impressions just as much as by the toxins of ordinary infective diseases. It would be interesting to know whether any difference in type is discoverable in the pyrexias produced under these different circumstances, or whether cross-circulation experiments would show a continuance of pyrexia in fevers if the central nervous system were supplied with pure blood. The question whether pyrexia is a remedial effort, or a mere consequence of disease more or less harmful to the patient, requires us to state whether all the organisms which produce the infective fevers are injured or otherwise by the high temperatures produced. Dr. Douglas Powell states[1] that unquestionably they do require for their cultivation a lower temperature, and that pyrexia tends to lower their activity. Hence remedies which lower the temperature below that normal to the disease may greatly increase the danger of microbic activity. Occasionally we find fevers running their course with subnormal temperatures. Teissier recently[2] related a case of pneumonia of this kind, and instances of typhoid and scarlatina are not unknown. In these few instances there may have been individual idiosyncrasies like the permanent subnormal temperatures occasionally found in healthy individuals, or some special compensation. When the temperature in typhoid is kept down to approximately the normal of health by continuous bathing, we ought to see whether or no microbic activity is increased. Though even here it might be replied that the test is not a sound one, for cold bathing acts largely by increasing the elimination of toxins, and not merely by reduction of temperature. It would seem then of great importance to determine by direct experiment for each kind of fever what is the temperature at which the activity of the special microbe is at its height.

Hale White,[3] in propounding the nervous origin of pyrexia, holds that it is due either to direct interference with the nervous centres, to reflex stimulation of them, or to the circulation through them of toxins. Thus experimental injury of the corpus striatum produces pyrexia, and it may be noted that a lesion of one only of these bodies gives a higher temperature on the opposite side of the body. Cortical lesions produce a highly irregular form of pyrexia, and hence some observers place the

[1] *Med. Week*, vol. ii., 1894, p. 376. [2] *N. York M. J.*, vol. lix., 1894, p. 287.
[3] *Med. Week*, vol. ii., 1894, p. 181.

thermotaxic mechanism in the latter, and the thermogenetic centres in the corpus striatum. The actual production of heat is in the muscles, and the thermolytic mechanism of the skin and lungs is under the control of a third special centre. Instances of reflex pyrexia occur in 75 per cent. of simple fractures, in biliary colic, and in aseptic burns; those caused by toxins of fevers are of every day occurrence, while the results of direct interference are seen in the pyrexia of meningitis, hysteria, chorea, and head or spinal injuries; the highly irregular temperature of meningitis being a typical case of cortical irritation. In short, every form of pyrexia can be traced in some way or other to influences acting on the central nervous system.

Professor Bouchard[1] illustrated a similar theory at the International Congress by quoting instances where emotion in weakly persons produces a rise in temperature. Such cases are seen every "visiting day" in our hospitals. Thus when the nervous system is debilitated the effect of the ordinary forces which produce or get rid of heat is exaggerated and we may have pyrexia from emotion, from sitting up in bed, or even from intellectual effort. He believes that ordinary muscular effort would produce a great rise of temperature if this was not controlled automatically by the thermotaxic mechanism in health. In the Bristol discussion there was general agreement as to the undesirableness of controlling moderate pyrexia. In hyperpyrexia, on the other hand, there is no doubt of the need of control, and the great question is how this may be best done. Two classes of antipyretics are sometimes open to our choice. In the one we find the drug acts as a direct poison to the microbes, thus cutting off the source of the fever; and in the other the sole result is that the pyrexia is got rid of without any action on the disease. Unfortunately there are few diseases where the former class are available, and we have therefore remaining the so-called new antipyretics, or quinine and cold baths. There are numerous plans of applying cold less clumsy than the bath. Dr. Curnow recommends an ice pack.[2] A sheet is wrung out in cold water, folded in four layers; pounded ice is placed between them, and the sheet wrapped round the patient until the temperature has fallen to 102°, but not afterwards. Another plan consists in placing a large rubber sheet under the patient, raising the edges all round with sandbags and bolsters, and then filling it with water, which can be siphoned off afterwards. Maillart, Lichtheim and others administer 5 or 6 litres of water daily by the mouth. Da Costa, Sciolla, Friedenwald,[3] and many French and American physicians have brought down the temperature by painting on the abdomen or chest 30 drops of pure guaiacol, and covering it over with waxed paper. The action is slow, but decided and lasting; but there is apt to be

[1] *Med. Week*, vol. ii., 1894, p. 157. [2] *Clin. J.*, vol. iii., 1894, p. 289.
[3] *N. York M. J.*, vol. lix., 1894, p. 455; *Practitioner*, vol. lii., 1894, pp. 289, 372.

much depression, and therefore the amount mentioned should not be exceeded at first.

Burney Yeo[1] claims that the results of wide experience under different observers show that his method obviates the need of baths or antipyretics almost entirely. He seeks not only to disinfect the intestinal canal, but also to destroy the toxins in the blood. He employs a mixture containing free chlorine and quinine every two or three hours, gives diluted milk or clear soup, and restricts the quantity if undigested. He finds that infusion of coffee is a better stimulant than alcohol in protracted cases, and praises Maillart's practice of administering great quantities of water to wash out the intestinal canal.

The treatment of various infective diseases by antitoxic serum, or dried antitoxins, has steadily progressed of late. Pneumonia, cholera, diphtheria, and tetanus have all been treated with considerable success. The first successful case of tetanus so treated in England is recorded from Leicester,[2] another is given by Dr. Herbert L. Evans from Goring, and a large number are related from continental sources. Haffkine's cholera treatment has already given promise of success in the escape of numbers of protected patients during epidemics. In typhoid the results are still doubtful, though Fränkel, Rumpf and others have used it largely. J. S. Ely[3] gives an interesting review of the reported cases of pneumonia treated in this way. Decided benefit seems to have followed, and very early defervescence. The regularity with which the critical symptoms followed upon the injections after an almost uniform interval of time seems to us, however, very forcibly to suggest a relationship of cause and effect, and at all events the harmlessness of the treatment is demonstrated.

The most important advance, however, is probably the final differentiation of true and false diphtheria, or that which contains Loeffler's bacillus and those forms which do not, and the treatment of the former by Aronson's antitoxin. Kaltz[4] reports from the Friedrich Hospital that 128 children were there treated by it this year with a mortality of only 13·2 per cent. They now inject as much as five drachms of the fluid, and have never found any bad results from its use, which was of course confined to those having the Loeffler bacillus, and in whom therefore the prognosis was serious. Six successful cases have quite recently been reported in England, treated with antitoxin supplied by Messrs. Zimmermann, Cross Lane, E.C., but the available stock is temporarily exhausted. The production of immunity by the same substance when administered to healthy persons throws much light on the physiology of its action.

[1] *Am. J. M. Sc.*, vol. cvii., 1894, p. 640. [2] *Lancet*, 1894, vol. i., p. 206.

[3] *Am. J. M. Sc.*, vol. cvii., 1894, p. 356.

If we compare the position and results of antitoxin treatment in these diseases with that attained twelve months ago, we must recognise that it has at last entered the range of practical medicine, in spite of many difficulties in application, and is yielding results at least equal to those given by other methods.

George Parker.

